# Ursachen und Konsequenzen von Niedrigzinsen

**DOI:** 10.1007/s41471-020-00099-w

**Published:** 2020-11-12

**Authors:** Benjamin Grosse-Rueschkamp, Jörg Rocholl

**Affiliations:** European School of Management and Technology, Schloßplatz 1, 10178 Berlin, Deutschland

**Keywords:** Geldpolitik, Natürlicher Zinssatz, Sparen, Risikofreie Anlagen, Finanzstabilität, Monetary policy, Natural rate of interest, Saving, Safe asset, Financial stability, E00, E40, E44, G00, G28, G50

## Abstract

Die anhaltende Niedrigzinsphase stellt Wirtschaft und Gesellschaft vor große Herausforderungen und wird daher in Wissenschaft und Öffentlichkeit intensiv diskutiert. Dieser Beitrag zeichnet die internationale wissenschaftliche Debatte nach und fasst die verschiedenen Argumente und empirischen Erkenntnisse zusammen. Nominal- und Realzinssätze sind seit mehr als drei Jahrzehnten global rückläufig und entziehen sich damit vermeintlich einfachen geldpolitischen Erklärungsansätzen. Vielmehr ergeben sich strukturelle Erklärungsansätze als wesentlicher Treiber dieser Entwicklung. Darüber hinaus zieht dieser Beitrag Schlüsse aus den empirischen Erkenntnissen und leitet daraus potentielle Konsequenzen sowie Handlungsempfehlungen für unterschiedliche ökonomische Akteure ab. Diskutiert werden Folgen insbesondere für die Übertragungsmechanismen der Geldpolitik, für Finanzintermediäre, für die Unternehmen der Realwirtschaft sowie für Politik und Haushalte.

## Abstract (en)

The sustained low-interest rate environment raises several economic challenges. These challenges as well as the underlying causes are subject to an intense economic debate. This article, traces the scientific debate by laying out the theoretical and empirical arguments. As not only nominal, but also real rates have decreased in most developed economies over a long period of time, monetary policy is unlikely to be the major driver of the development. In contrast, theories that explain the decline in interest rates with structural changes in the demand and supply of capital are consistent with the empirical evidence and offer a more convincing explanation. Beyond an analysis of the roots of the decrease, we discuss potential economic consequences of persistently low interest rates. In particular, we discuss evidence of how the transmission channels of monetary policy, financial intermediaries, non-financial companies as well households are affected by the low-interest rate environment. Finally, we provide policy recommendations.

Eines der wichtigsten makro- und finanzökonomischen Phänomene unserer Zeit ist die seit mehreren Jahren anhaltende Niedrigzinsphase. Aufgrund der hohen Bedeutung des Themas für Politik, Wirtschaft und Gesellschaft wird das Thema sowohl in der Öffentlichkeit als auch in Fachkreisen breit debattiert. Da sich das Phänomen einer monokausalen Erklärung entzieht und klare Evidenz für einzelne Erklärungsansätze aussteht, wird die Debatte teilweise sehr kontrovers geführt.^1^

Dieser Aufsatz hat das Ziel, die Diskussion über die Ursachen der anhaltenden Niedrigzinsphase nachzuzeichnen und eine Übersicht über die Argumente zu geben. Darüber hinaus versucht der Text, Schlüsse aus den bisherigen Erkenntnissen zu ziehen und potenzielle Konsequenzen und Handlungsempfehlungen für unterschiedliche ökonomische Akteure, von Privatanlegern bis zu politischen Entscheidern, aufzuzeigen.

Dieser Artikel ist folgendermaßen strukturiert: im ersten Abschnitt wird eine Bestandsaufnahme durchgeführt, in der Fakten deskriptiv dargelegt werden. Die Kernbotschaft dieses Abschnitts lautet, dass es sich bei Niedrigzinsen um einen längerfristigen und globalen Trend handelt. Im zweiten Abschnitt wird die Rolle der Geldpolitik diskutiert. Die klassische makroökonomische Sichtweise betrachtet die Wirkungen der Geldpolitik in der langen Frist neutral, was die Zentralbankpolitik als potenziellen Verursacher exkulpiert. Wir geben Argumente wieder, denen zufolge dieses Narrativ möglichweise unvollständig ist. Im dritten Abschnitt geht es um die Rolle struktureller Faktoren. Hier diskutieren wir, welche Faktoren zu einem Rückgang in der Kapitalnachfrage bzw. zu einem Anstieg im Kapitalangebot geführt haben könnten und dadurch potenziell für fallende Zinssätze verantwortlich sind. Darunter fallen Faktoren wie die Demographie, Sparquoten in Schwellenländern, steigende Marktkonzentration, fallende relative Preise von Investitionsgütern und steigende Ungleichheit. Darüber hinaus wird auf die besondere Rolle von risikofreien Anlagen eingegangen.

Darauf folgt eine Diskussion zu den Konsequenzen aus Niedrigzinssätzen. Wir betrachten dort zuerst die Konsequenzen für die Geldpolitik und die Finanzwirtschaft. Die Hauptaussage lautet, dass insbesondere beim Erreichen der Nullzinsgrenze der Übertragungsmechanismus der Geldpolitik und die Finanzstabilität zunehmend gefährdet sind. Es folgen Betrachtungen zur Situation der Realwirtschaft sowie der Haushalte und Politik. Eine der zentralen Schlussfolgerungen dieses Abschnittes lautet, dass insbesondere deutsche Haushalte Portfolioumschichtungen zugunsten renditestärkerer Anlageformen vornehmen sollten. Schließlich fassen wir die wichtigsten Erkenntnisse zusammen.

## Einleitung und Bestandsaufnahme

**Figure 1:**
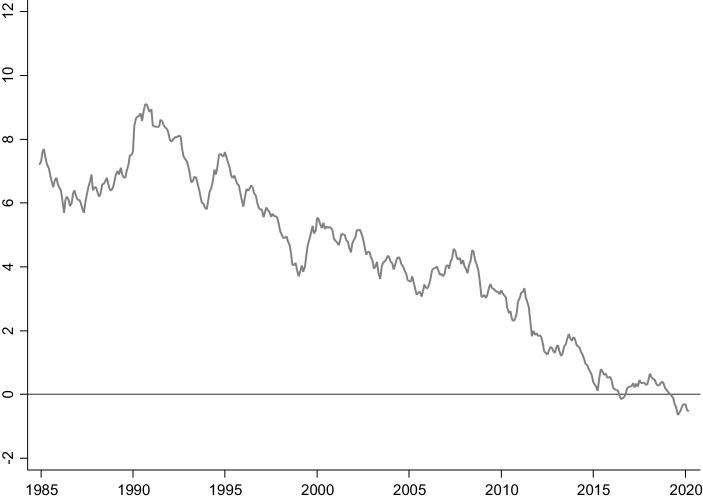

Seit den 1980er-Jahren ist der reale Zinssatz, d. h. der Zinssatz nach Berücksichtigung der Inflation, gesunken. Gleichzeitig hat eine langanhaltende Phase niedriger Inflationsraten eingesetzt; demnach sind auch die nominalen Zinssätze gefallen. In Abb. 1 ist die Rendite der langfristigen Staatsanleihen des Bundes im Zeitverlauf abgetragen. Die Bundesanleihen besitzen im Euroraum sogenannten Benchmark-Status, d. h. aufgrund der hohen Sicherheit und Marktliquidität der Anleihen gilt ihr jeweils aktueller Handelskurs auf den Finanzmärkten als wichtiger Orientierungspunkt. Aus diesem Grund bildet die sich aus dem Kurs resultierende Rendite einen der wichtigsten Zinssätze im Euroraum und darüber hinaus. Aus dem Verlauf der Rendite in Abb. 1 ist ersichtlich, dass diese Rendite seit Mitte der 1980er-Jahre im langfristigen Durchschnitt gefallen ist.

**Figure 2:**
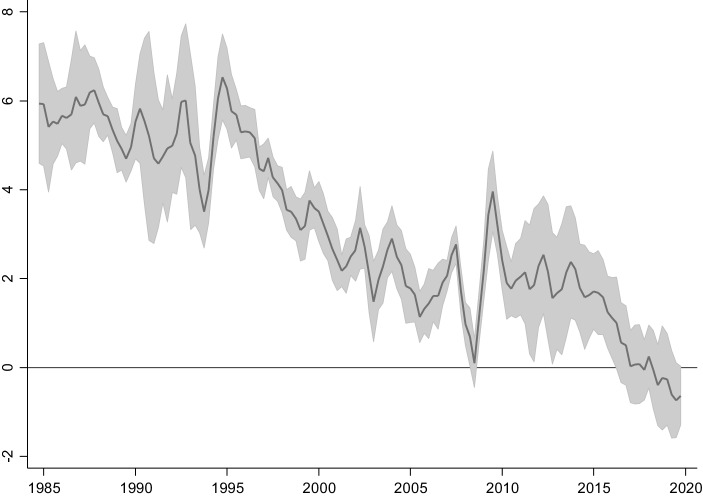

Diese Entwicklung ist nicht auf Deutschland oder die Eurozone beschränkt, sondern stellt ein globales Phänomen dar. Abb. 2 zeigt einen Rückgang im durchschnittlichen realen Zinssatz der Industrieländer von ca. 6 % in den 1980er-Jahren, auf unterhalb von 0 % Ende der 2010er-Jahre. Darüber hinaus wird aus Abb. 2 ebenfalls ersichtlich, dass sich die Abweichung der Zinssätze zwischen den Volkswirtschaften verringert hat, d. h. der Rückgang des mittleren Realzinses wird nicht durch einige Ausreißer getrieben, sondern ist eine Entwicklung, die in vergleichbarem Ausmaß alle Industrieländer betrifft. Dies wird in Abb. 2 durch den Interquartilsabstand dargestellt, der die Streuung der Zinssätze der verschiedenen Länder um ihr gemeinsames arithmetisches Mittel zu jedem Zeitpunkt anzeigt. Diese Streuung ist ebenso wie der Mittelwert in dem angegebenen Zeitraum geschrumpft, wenngleich die Streuung der Renditen vor allem während der Eurokrise aufgrund der schwachen Fiskalposition einiger Länder wieder angestiegen ist. Die tendenzielle Vereinheitlichung der Zinssätze lässt Blanchard et al. (2014) von einem zunehmend „globalen Zinssatz“ sprechen, der auf einem globalen Markt gebildet werde. Risikoadjustierte Unterschiede im Zinssatz zwischen den unterschiedlichen Volkswirtschaften werden auf einem solchen Markt durch Arbitrage durch sogenannte Carry-Trades^2^ im Extremfall vollständig eliminiert.

Während der Befund, dass es sich um eine globale Entwicklung handle, weitgehend anerkannt wird, ist in den letzten Jahren eine oft kontroverse Diskussion um die Ursachen des Rückganges geführt worden. Da es sich um einen längerfristigen Trend handelt und sich der reale Zinssatz als Durchschnitt über die jeweiligen Konjunkturzyklen hinweg bei jedem folgenden Zyklus weiter verringert hat (Krugman 2014), ist die Erklärung als ein rein zyklisches Phänomen unwahrscheinlich, obgleich zyklische Faktoren, vor allem in den Jahren der Finanzkrise von 2008, einen verstärkenden Effekt gehabt haben könnten.

## Die Rolle der Geldpolitik

Der Einfluss der Geldpolitik wird kontrovers diskutiert. Klassischen makroökonomischen Modellen zufolge wirkt Geldpolitik zumindest in der langen Frist neutral, d. h. Zentralbankpolitik hat lediglich einen Effekt auf *nominale* Variablen, während *reale* Variablen hingegen nicht dauerhafte beeinflusst werden können (siehe z. B. King und Watson 1997). Die Beobachtung der gefallenen realen Renditen hat viele Ökonomen schließen lassen, dass der sogenannte natürliche Zinssatz gefallen ist (Summers und Rachel 2019). Der natürliche Zinssatz entspricht dem Niveau des kurzfristigen realen Gleichgewichtszinssatzes, bei dem gesamtwirtschaftlich die tatsächliche Produktion der potenziellen Produktion entspricht und der sich in einer Volkswirtschaft mit vollständig flexiblen Preisen bei Vollbeschäftigung bilden würde. In diesem Zustand herrscht eine konstante Inflationsrate. Im Falle einer dauerhaft zu expansiven Geldpolitik, die den Zentralbankzins auch in Zeiten, in denen die Ökonomie an ihrer Potentialgrenze operiert, zu niedrig belässt, würde die Inflation steigen. Eine Implikation dieser Sichtweise ist, dass bei konstanter Inflation Geldpolitik dem natürlichen Zinssatz mehr oder weniger passiv folgt. Dass die Inflation der letzten Jahrzehnte konstant niedrig gewesen ist und im letzten Jahrzehnt regelmäßig sogar unterhalb der von Zentralbanken ausgegebenen Inflationsziele lag, stützt das Narrativ des fallenden natürlichen Zinssatzes deutlich. Dieses steht im direkten Widerspruch zur These, dass niedrige Zinssätze zuvorderst ein Resultat übertrieben expansiver Geldpolitik seien, eine in der deutschen Politik und Presseberichterstattung häufig geäußerte Ansicht.

Nichtdestotrotz gibt es Argumente dafür, dass das gerade beschriebene Narrativ möglicherweise unvollständig ist. Autoren der Bank für Internationalen Zahlungsausgleich (Juselius et al. 2016) zufolge gibt es Anhaltspunkte, dass die Geldpolitik durch ihren Einfluss auf finanzielle Variablen dauerhaften Einfluss auf das Potentialwachstum und dementsprechend auf den natürlichen Zinssatz haben könnte. Und zwar argumentieren die Autoren, dass die Geldpolitik die durchschnittliche Schuldendienstbelastung einer Ökonomie beeinflusst, die durch den Verschuldungsgrad und die Vermögenspreisentwicklung im Zusammenhang mit der Potentialproduktion steht. Die Autoren fordern, dass Zentralbanken eine sehr viel deutlichere Geldpolitik des „Sich-gegen-den-Wind-Lehnens“ durchführen sollten, um damit das Kredit- und Vermögenspreiswachstum durch Zinserhöhungen im Aufschwung frühzeitiger zu begrenzen. Andernfalls würde expansive Geldpolitik sich dauerhaft verstetigen, da ein starker „Boom-Bust-Zyklus“ das Potentialwachstum beschädige und in der überschuldeten Wirtschaft eine kontinuierlich expansive Geldpolitik nötig mache.

Als weiterer Punkt wird diskutiert, inwieweit durch das Zusammenspiel von expansiver Geldpolitik und einem unterkapitalisierten Bankensektor Liquidität an nicht mehr kompetitive Unternehmen geleitet wird, deren Insolvenz dadurch lediglich hinausgezögert, aber letztlich nicht vermieden wird. Durch diese sogenannten Zombie-Kredite können in den betroffenen Branchen Überkapazitäten entstehen, die bei gleichzeitig fallender durchschnittlicher Produktivität und sinkenden Investitionen zu sinkenden Produzentenpreisen führen können (Acharya et al. 2019a). Die dadurch entstehenden deflationären Tendenzen würden zu fallenden Zinsen beitragen.

Entgegen der Intuition klassischer makroökonomischer Modelle argumentieren einige Ökonomen, dass eine Zentralbank an der Nullzinsgrenze durch Zinsanhebungen Inflation erzeugen kann, dadurch der Liquiditätsfalle entkommt und so auf einen normalen Zinspfad zurückkehren kann. Dieser Effekt hängt jedoch davon ab, dass die Zinssatzanhebung der Zentralbank glaubwürdig als dauerhafte Selbstverpflichtung wahrgenommen wird. Dieser Mechanismus wird auch als Neo-Fisher Effekt bezeichnet und wird als potenzieller Ausweg aus dem Szenario niedriger Zinsen bei gleichzeitig niedriger Inflation diskutiert (Uribe 2020).

Obschon aktuelle wissenschaftliche Arbeiten an der Identifizierung bisher unbekannter Wechselwirkungen zwischen Geldpolitik und realen Variablen arbeiten, ist das Narrativ von Zentralbanken als Hauptverantwortliche für niedrige Zinsen nicht mit dem bereits seit mehr als drei Jahrzehnten global fallenden Zinssätzen bei gleichzeitig niedriger Inflation vereinbar und scheidet als wichtigster Erklärungsansatz aus. Im Folgenden werden daher strukturelle Faktoren als potenzielle Ursachen beleuchtet.

## Die Rolle Struktureller Faktoren

Da der Zinssatz, oder allgemeiner, die Kapitalrendite, als Mietpreis für Kapital verstanden werden kann, ist es naheliegend, die Ursachen des fallenden Zinssatzes in der Kapitalangebots- oder -Nachfrageseite zu suchen. Wenn aus strukturellen Gründen das Kapitalangebot zunimmt und/oder die Kapitalnachfrage zurückgeht, dann muss der Zinssatz ebenfalls fallen, um die Kapitalmärkte wieder ins Gleichgewicht zu bringen. Demnach lässt sich ein fallender Zinssatz mit einem Anstieg im Kapitalangebot, einem Rückgang in der Kapitalnachfrage oder beiden Entwicklungen erklären.

Dem Konzept der säkularen Stagnation zufolge (Summers 2014) führt das gestiegene Kapitalangebot und die gefallene Kapitalnachfrage zu einer Situation, in der ein Angebotsüberhang an Kapital die gesamtwirtschaftliche Nachfrage reduziert, das Wirtschaftswachstum und die Inflation dämpft und dadurch die realen Zinssätze fallen lässt. In einem solchen Szenario besteht die Möglichkeit, dass der natürliche Zinssatz negativ ist. Aufgrund der Nullzinsgrenze kann also der Zinssatz, der nötig wäre, um den Markt für Kapital bei Vollbeschäftigung zu räumen, nicht erreicht werden. Das wiederrum kann laut Summers (2014) zu Problemen wie längerfristigem Niedrigwachstum und Vermögenswertblasen und daraus resultierender Finanzinstabilität führen.

Auch wenn nicht geklärt ist, ob sich die Welt tatsächlich in einem Szenario der säkularen Stagnation befindet, so bleibt die Grundthese, dass Änderungen im Kapitalangebot und -Nachfrage für den fallenden Zinssatz (mit-)verantwortlich sind, schlüssig.

### Anstieg des Kapitalangebotes

Das Kapitalangebot in einer Volkswirtschaft wird durch Sparer im Austausch gegen Forderungen, d. h. Eigentums- oder Schuldtitel bereitgestellt. Diese Forderungen können direkt gehalten werden, beispielsweise in Form von Anleihen, Immobilien- bzw. Unternehmenseigentum, oder indirekt durch Finanzintermediäre wie Banken, Fonds oder Versicherungen. Das Kapitalangebot ergibt sich aus den Ersparnissen der Haushalte, d. h. für das absolute Angebot ist einerseits die Sparquote als Anteil der Ersparnisse am Einkommen und andererseits die Höhe des Einkommens relevant; in der Regel wird jedoch hauptsächlich die relative Größe der Sparquote betrachtet. Insofern die Kapitalmärkte weltweit als integriert betrachtet werden können und damit nationale Zinssatzdifferenzen durch Kapitalströme ausgeglichen werden können, sind weniger die nationalen Sparquoten als der nach Wirtschaftsgröße gewichtete Durchschnitt der Sparquoten aller Länder für die Betrachtung des Kapitalangebots relevant. Trotz vieler weiterhin bestehender Friktionen zeigt die Konvergenz der nationalen Zinssätze, wie in Abb. 2 zu erkennen, dass die Annahme eines global integrierten Marktes eine gute Annäherung an die Realität darstellt. Dementsprechend werden im Folgenden mehrere Entwicklungen der letzten Jahrzehnte diskutiert, die potenziell eine Erhöhung der globalen Sparquote bewirkt haben.

Ein potenzieller Grund für einen Anstieg im Kapitalangebot ist in den Auswirkungen des demographischen Wandels zu suchen, der in unterschiedlichem Ausmaß sowohl Industrie- als auch Schwellenländer betrifft. Optimales Verhalten für rationale Agenten in klassischen Lebenszyklusmodellen setzt eine Vermögensaufbauphase voraus, die zeitlich mit dem Arbeitsleben zusammenfällt. Da das Einkommen im Allgemeinen mit der Berufserfahrung steigt, sind die höchsten Einkommen und damit Ersparnisse gegen Ende des Berufslebens zu erwarten. Im Ruhestand findet dann ein Entsparen statt, dies entspricht einer negativen Sparquote, und das zuvor akkumulierte Vermögen wird graduell aufgebraucht. Der demographische Wandel ist ein Zusammenspiel zweier Entwicklungen, zum einen der steigenden Lebenserwartung und zum anderen der fallenden Fertilität. So trägt eine steigende Lebenserwartung, die zu der berechtigten Erwartung einer erheblichen Lebenszeit im Ruhestand führt, zu einem wachsenden Bedarf an Altersvorsorge bei. Zusammen mit einem Kohorteneffekt bietet sie eine potenzielle Erklärung für den sinkenden Realzinssatz: die geburtenstarken Jahrgänge der Nachkriegsgeneration, die in den 1970er und 1980er-Jahren in das Berufsleben eingestiegen ist, gehen auf den Ruhestand zu. Gemäß dem Lebenszyklusmodell ist demnach seit dem Berufseinstieg ein zunehmend höheres Kapitalangebot aufgrund steigender Sparleistung zu erwarten gewesen. Dieses Verhalten könnte auch durch Unsicherheit bezüglich der Leistungsfähigkeit von staatlich organisierten Altersvorsorgeprogrammen verstärkt worden sein, insbesondere bei umlagebasierten Systemen, die durch den demographischen Wandel zunehmend in eine Schieflage geraten.

Schwellenländer sind in den letzten 30 Jahren immer stärker in das internationale Handelssystem und die internationalen Kapitalmärkte integriert worden. Zuvorderst China, aber auch Länder wie Russland, Brasilien oder Saudi-Arabien, weisen im Durchschnitt sehr hohe gesamtwirtschaftliche Sparquoten auf. Entwicklungs- und Schwellenländer haben aufgrund der geringen Kapitalintensität ihrer Volkswirtschaft einen hohen Investitionsbedarf. Die Sparquoten in den Schwellenländern liegen allerdings so hoch, dass sie die sehr hohe inländische Investitionsquote übersteigen. So liegt beispielsweise die Sparquote in China bei 46 % des Bruttoinlandsprodukts (Weltbank 2018). Die positive Differenz zwischen Spar- und Investitionsquote führt zu einem Leistungsbilanzüberschuss, der das Nettokapitalangebot auf den globalen Finanzmärkten erhöht und damit eine sogenannte Sparschwemme verursacht (Bernanke 2005). Ein Grund für die hohe gesamtwirtschaftliche Sparquote könnte im Aufbau von Devisenreserven vieler Schwellenländer als Vorsichtsmaßnahme nach den Erfahrungen in den Währungskrisen der 1990er-Jahren liegen, wie in Mexiko 1994, Ostasien 1997–98, und Russland, Brasilien und Argentinien 1998, 1999 und 2002. Für diese These spricht, dass die Schwellenländer Mitte der 1990er-Jahre noch Kapitalimporteure waren und erst in den letzten Jahren des letzten Millenniums zu Kapitalexporteuren wurden. Zugleich gibt es die These, dass das Fehlen von zuverlässigen Sozialsystemen Haushalte in Schwellenländern zu Vorsichtssparen motiviert. Dieser Faktor würde bei steigenden Einkommen quantitativ relevanter werden, da viele Haushalte mit dem Überschreiten der Subsistenzschwelle zum ersten Mal die Möglichkeit zum Sparen bekommen und ein prozentual hoher Anteil des steigenden Einkommens zurückgelegt wird.

Ein weiterer Trend seit den 1980er-Jahren, insbesondere in den USA, ist eine steigende Einkommensungleichheit (Piketty et al. 2018). So lag im Jahr 2014 das durchschnittliche Vorsteuereinkommen in der unteren Hälfte der Einkommensverteilung in den USA bei ca. USD 16.000 pro erwachsenen Amerikaner und ist kaufkraftbereinigt seit 1980 nicht mehr gestiegen. Auch die Berücksichtigung von Steuern und Transfers ändert den Befund nur marginal. Gleichzeitig ist das Durchschnittseinkommen aller Amerikaner im selben Zeitraum um 60 % auf USD 64.500 gestiegen, was im Ergebnis dazu führte, dass der Einkommensanteil der unteren Hälfte von 20 % in 1980 auf nur 12 % in 2014 gefallen ist. Das Bild wird noch deutlicher, wenn man den obersten Rand der Verteilung betrachtet: so ist das jährliche Vorsteuereinkommen der bestverdienenden 1 % der amerikanischen Bevölkerung von USD 420,000 in 1980 auf USD 1,3 Mio. im Jahr 2014 angestiegen. Diese Entwicklung ist in Europa deutlich weniger eindeutig als in den USA, vor allem bei Betrachtung der Einkommen nach Steuern und Transfers in Deutschland, aber ein zumindest leichter Trend zu mehr Einkommensungleichheit lässt sich in einigen europäischen Ländern ebenso feststellen (Alvaredo, Atkinson, Picketty, Saez und Zucman, 2011–2017; Wissenschaftlicher Beirat beim BMF 2017). Wenn reichere Haushalte im Durchschnitt einen höheren Anteil ihres Einkommens sparen, dann führt ein Anstieg der Einkommen im oberen Teil der Einkommensverteilung zu steigenden Ersparnissen insgesamt. Sowohl die gestiegene Einkommensungleichheit als auch die im Durchschnitt höhere Sparquote reicher Haushalte sind gut belegt. Mian et al. (2020) zeigen Evidenz, der zufolge der Anstieg der Ersparnisse in den USA durch die Einkommensungleichheit in der gleichen Größenordnung liegen wie die zuvor beschriebene internationalen Sparschwemme. So reichen laut Mian et al. (2020) seit dem Jahr 2000 die jährlichen Ersparnisse der reichsten 1 % der Amerikaner aus, den Mittelbedarf der Nettoinvestitionen der US-amerikanischen Wirtschaft zu decken.

### Abnahme der Kapitalnachfrage

Es gibt nicht nur Hinweise auf ein gestiegenes Kapitalangebot, sondern zugleich ist es möglich, dass die Nachfrage nach Kapital gefallen sein könnte. Hierzu werden die folgenden potenziellen Ursachen diskutiert.

Der bereits diskutierte Trend der steigenden Lebenserwartung ist nicht der einzige Aspekt, der den demographischen Wandel antreibt. Zugleich ist auch die Geburtenrate gefallen und befindet sich in einigen Ländern, darunter in Deutschland, unterhalb des Reproduktionsniveaus von 2,1 Kindern pro Frau. Dies hat Konsequenzen für die Größe der Erwerbsbevölkerung, die in vielen Industrieländern nur noch durch Migration konstant gehalten werden kann. Durch den sich daraus ergebenden stagnierenden oder fallenden Anteil der Erwerbsbevölkerung sowie durch eine bereits jetzt hohe Kapitalintensität sinkt die Anzahl der profitablen Investitionsmöglichkeiten. Dies verringert die Nachfrage nach Kapital.

Als weiterer Punkt wird häufig der Rückgang der relativen Preise von Kapitalgütern erwähnt. Seit 1990 sind die Preise von Maschinen und Anlagen relativ zu Konsumgüterpreisen um 40–60 % gefallen. Ein besonders herausragendes Beispiel ist der Rückgang der Preise von Computern in Höhe von 90 % in diesem Zeitraum. Computer sind ein wichtiger Inputfaktor in vielen Geschäftsmodellen. Niedrigere Preise von Kapitalgütern haben das Potenzial, die Kapitalnachfrage zu senken, wenn eine identische Investitionshöhe mit geringeren Ausgaben erreicht werden kann. Zugleich ist es jedoch plausibel, dass geringere relative Preise von Kapitalgütern die optimale Investitionshöhe gegenüber dem Ausgangszustand erhöht haben (Lian et al. 2019). Dieser Effekt würde den Rückgang der relativen Kosten von Kapitalgütern durch einen Anstieg der Nachfrage derselben zu einem gewissen Grad kompensieren. Der Nettoeffekt geringerer Preise von Investitionsgütern auf die Kapitalnachfrage ist daher umstritten.

Darüber hinaus ist die Bedeutung von physischen Produktionsfaktoren, beispielsweise Maschinen und Fabriken, gegenüber immateriellen Produktionsfaktoren wie geistiges Eigentum, d. h. Patente, Software, Markenrechte etc., gefallen. Die Bereitstellung von immateriellen Produktionsfaktoren erfordert üblicherweise einen hohen Einsatz an Humankapital und einen geringeren Kapitaleinsatz. Zudem sind physische Kapitalgüter aufgrund ihrer besseren Eigenschaften als Kreditsicherheiten leichter zu finanzieren. Diese Gründe könnten weiterhin zum Rückgang der Kapitalnachfrage beigetragen haben.

Ein weiterer wichtiger makroökonomischer Trend ist eine abnehmende Wettbewerbsintensität und steigende Marktkonzentration seit den 1980er-Jahren, speziell in den USA. Dieser Effekt kann auch auf das steigende Gewicht sogenannter Superstar-Firmen zurückgeführt werden (Autor et al. 2020). Superstar-Firmen sind hochproduktive Unternehmen, die in ihrer jeweiligen Branche unangefochtene Marktführer sind und in ihren Geschäften hohe Margen erzielen können. Die steigende Marktkonzentration wurde in vielen Fällen durch Innovationen getrieben, die die durch hohe Fix- und niedrige variable Kosten charakterisierten softwaregetriebenen Geschäftsmodelle ermöglichen. Dies sorgt für die wachsende Bedeutung von Skaleneffekten, die im Falle der Plattform-Ökonomie auf die Spitze getrieben werden und zu einer extremen Konzentration führen. Skaleneffekte haben die Folge, dass der Anteil der Wertschöpfung der produktivsten Firmen in einer „Winner-takes-most“-Dynamik steigt, was zu steigender Marktmacht führt. Diese erlaubt den Marktführern, ihre Margen zu erhöhen. So haben sich die aggregierten prozentualen Preisaufschläge über Grenzkosten, die zwischen den 1950er-Jahren und 1980 konstant gewesen sind, seitdem verdreifacht, während die fixen Betriebskosten in weit geringerem Ausmaß gestiegen sind (De Loecker et al. 2020). Zwar ist der Zusammenhang zwischen Wettbewerb und Investitionen theoretisch nicht eindeutig, empirische Studien haben aber Evidenz gefunden, dass die gestiegene Marktkonzentration zu niedrigeren Investitionen geführt hat, und damit zur fallenden Kapitalnachfrage beigetragen hat (Gutiérrez und Philippon, 2017). Ökonomen haben versucht, die Auswirkungen der gestiegenen Marktkonzentration auf die Kapitalnachfrage zu quantifizieren. Basierend auf einem in der makroökonomischen Literatur üblichen DSGE-Modell simulieren Jones und Philippon (2016) eine kontrafaktische US-Ökonomie, in der die Wettbewerbsintensität auf dem Niveau des Jahres 2000 verblieben ist. Den Schätzungen zufolge hätte die US-Wirtschaft ohne den Wettbewerbsrückgang den Nullzinssatz mehrere Jahre früher hinter sich gelassen.

### Angebot und Nachfrage nach risikofreien Anlagen

In der vorhergehenden Diskussion wurde implizit von einer einheitlichen Wirkung von Veränderungen in Angebot und Nachfrage von Kapital auf unterschiedliche Arten der Verzinsung ausgegangen. Zinssätze oder allgemeiner Kapitalrenditen, unterscheiden sich beispielsweise in ihrer Laufzeit und in ihrem Risiko. Üblicherweise wird davon ausgegangen, dass unterschiedliche Renditen und Risiken durch Risikoprämien zueinander in Beziehung stehen. Die Risikoprämien, meistens als Aufschlag über dem risikofreien Zinssatz gemessen, kompensieren für die Übernahme von Risiken. Wenn sich also beispielsweise durch die bereits diskutierte „Sparschwemme“ das Angebot auf den Kapitalmärkten erhöht, wirkt sich dies im Prinzip auf sämtliche Kapitalanlagen aus, da risikoadjustierte Renditeunterschiede durch Arbitrageure ausgenutzt werden können und dadurch in der Regel verschwinden^3^. Dieser Mechanismus kann begründen, weshalb eine allgemeine Änderung im Angebot oder Nachfrage nach Kapital, wie in den vorherigen Abschnitten aufgeführt, ceteris paribus Renditen unterschiedlicher Anlageklassen betrifft.

Caballero, Farhi und Gourinchas (2017a) analysieren explizit die Entwicklung des risikofreien Zinssatzes und argumentieren, im Gegensatz zu den im Vorabsatz beschriebenen Überlegungen, dass die durchschnittliche Rendite auf Unternehmenskapital in den letzten Jahrzehnten stabil geblieben und lediglich die Rendite auf Staatsanleihen gefallen sei. Dies könnte implizieren, dass es weniger einen allgemeinen Anstieg im Kapitalangebot relativ zur Kapitalnachfrage gab, sondern viel mehr eine Verschiebung der Nachfrage von riskanten zu sicheren Anlageformen.

Dass die Kapitalkosten der Unternehmen^4^, die besser das relative Angebot an Kapital für den Unternehmenssektor wiederspiegeln, tatsächlich konstant geblieben seien, wird von anderen Ökonomen allerdings angezweifelt. So werden Eigenkapitalkosten von Unternehmen meistens mit Hilfe von realisierten Renditen geschätzt^5^. Da die realisierten Renditen aber zunehmend Kompensation für die Erschaffung immateriellen Kapitals und/oder ökonomische Renten durch Marktkonzentration enthalten, ergibt sich ein Keil zwischen der Rendite auf Unternehmenskapital, die konstant oder sogar gestiegen ist, und den tatsächlichen Kosten für Unternehmenskapital, die wiederum tendenziell gefallen sind (Crouzet und Eberly 2020; Barkai 2020).

Nichtdestotrotz scheint es plausibel zu sein, dass die Rendite auf risikofreie Anlagen, wie Staatsanleihen oder durch Einlagensicherungssysteme abgesicherte Bankeinlagen, im Vergleich zur Rendite auf riskantere Anlagen, wie Kredite oder Eigenkapital von Unternehmen, besonders stark gesunken ist. Dies könnte durch eine gestiegene Nachfrage nach risikofreien Anlagen bei gleichzeitigem relativem Rückgang derselben als Anteil am globalen Bruttoinlandsprodukt erfolgt sein (Caballero et al. 2017b).

Nur wenige Akteure in der globalen Ökonomie sind in der Lage, risikofreie Anlagen zu generieren, hauptsächlich Staaten und Finanzintermediäre einiger weniger Industrieländer. Der Anzahl dieser Akteure ist jedoch insbesondere seit der Finanzkrise von 2008 und der darauffolgenden Eurokrise gefallen. Eine Studie der Barclays Bank (Barclays 2012) schätzt den Rückgang der sicheren Anleihen von 37 % des Welt-BIPs in 2007 auf nur 18 % in 2011. Die Entwicklung wurde getrieben durch eine Neubewertung des Kreditrisikos von verbrieften Wohnungsbaukrediten in den USA sowie der Staatsanleihen einiger europäischer Staaten, die nicht mehr als risikofrei wahrgenommen werden.

Dem gegenüber steht eine gestiegene Nachfrage nach sicheren Anlagen. Diese stammt, wie oben ausgeführt, zum einen aus einer gestiegenen Sparneigung verschiedener Akteure. Diese möchten ihre Ersparnisse in Zeiten hoher makroökonomischer Volatilität in sicheren und vor allem auch liquiden Wertpapieren anlegen. Zum anderen sind insbesondere im Nachgang der Finanzkrise 2008 neue Regulierungen erlassen worden, die Banken erhöhte Liquiditätspolster vorschreiben, was zu einem weiteren Anstieg der Nachfrage nach sicheren Anlagen geführt hat. Der Rückgang im Angebot einerseits und der Anstieg der Nachfrage andererseits bietet eine Erklärung für den gefallenen risikofreien Zinssatz.

## Konsequenzen aus Niedrigzinsen

### Konsequenzen für Finanzstabilität und Geldpolitik

Mit sehr niedrigen Zinsen sind Herausforderungen für Finanzintermediäre und damit auch für den Transmissionsmechanismus der Geldpolitik verbunden. Das kann Folgen sowohl für die Finanzstabilität einer Ökonomie haben als auch für die Fähigkeit von Zentralbanken, ihre geldpolitischen Maßnahmen zu implementieren. So zeigt die empirische Evidenz, dass niedrige Zentralbankzinsen die Kreditvergabestandards der Geschäftsbanken aufweichen und dadurch eine riskantere Geschäftspolitik von Geschäftsbanken verursachen (Jiménez et al. 2014). Dies bestätigt Theorien, die erklären, inwieweit eine durch Geldpolitik ausgelöste Liquiditätserhöhung zu einer erhöhten Risikofreude bei Banken führen kann, wenn Banken aufgrund niedriger Kapitalisierung die Risiken potenzieller Kreditausfälle nicht vollständig internalisieren, was eine Form des Moral Hazard darstellt.

Niedrige Realzinsen und schwache Inflation implizieren, dass Zentralbanken mit ihrem Standardinstrument, dem kurzfristigen nominalen Zinssatz, immer häufiger durch die Nullzinsgrenze^6^ begrenzt werden. Dies verringert die Fähigkeit weiterer expansiver Geldpolitik. Damit ist das Erreichen eines Inflationsziels gefährdet, insbesondere, wenn Unternehmen und Haushalte durch anhaltend niedrige Inflation ihre Inflationserwartungen anpassen. Dazu ist es allerdings nötig, dass diese ihre Erwartungen zumindest teilweise vom gegenwärtigen Inflationsniveau beeinflussen lassen oder die Glaubwürdigkeit des Inflationsziels in Frage gestellt wird. Darüber hinaus, wenn Nominalzinssenkungen nicht mehr möglich sind, sehen Zentralbanken ihre Handlungsfähigkeiten bei neuen makroökonomischen Schocks reduziert.

Um in einem Umfeld deflationärer Tendenzen dennoch die Geldmenge zur Erreichung ihres Inflationsziels anheben zu können, haben sich Zentralbanken gezwungen gesehen, auf unkonventionelle Geldpolitik auszuweichen. So hat die Europäische Zentralbank eine Reihe von Instrumenten der unkonventionellen Geldpolitik angewandt, darunter negative Zentralbankzinsen, gezielte längerfristige Refinanzierungsgeschäfte (TLTRO), vorausschauende Zielsetzung (Forward Guidance), und Wertpapieraufkaufprogramme mit dem Ziel, auch beim Erreichen der Nullzinsgrenze weiterhin expansiv tätig sein zu können. In der gegenwärtigen Coronavirus-Krise hat die Europäische Zentralbank als Hauptinstrument ein neues Wertpapierkaufprogramm, das Pandemic Emergency Purchase Programme, aufgelegt.

Die Wirkungen dieser geldpolitischen Instrumente sind noch nicht vollständig erforscht. Insbesondere können sie auch unerwünschte Nebenwirkungen zeigen. Beispielsweise kommt es beim Aufkauf von Vermögenstiteln durch Zentralbanken im Rahmen der Quantitativen Lockerung für die Wirkung stark darauf an, welcher Sektor die Wertpapiere hält und welche Wertpapiere aufgekauft werden. So wurde Evidenz dafür gefunden, dass der Aufkauf hypothekenbesicherter Wertpapiere durch die amerikanische Federal Reserve Bank dazu geführt hat, dass Banken vermehrt Hypotheken refinanziert haben, dadurch aber ihre Kredite an Unternehmen verringert haben. Unternehmen, die von diesen Banken finanziert wurden, mussten ihre Investitionen reduzieren (Chakraborty et al. 2019).

Ein anderes Beispiel ist Evidenz für Zombie-Kredite von unterkapitalisierten Banken an überschuldete Unternehmen. Die Einführung des Outright Monetary Transaction-Programms, mit dem die Voraussetzungen für unbegrenzte Aufkäufe von Staatsanleihen durch die Europäische Zentralbank geschaffen wurden, hat zu starken Kursgewinnen bei Staatsanleihen der Peripherieländer geführt. Dies hat bei Banken, die viele solcher Anleihen in ihren Büchern hatten, zu einem unerwarteten Einmalgewinn geführt. Die dadurch entstehenden Möglichkeiten zur Kreditvergabe wurden dann aber teilweise dazu genutzt, überschuldeten Unternehmen Kredite zu gewähren (Acharya et al. 2019b). Dies hat über die folgenden Jahre zu verringerten Investitionen und Beschäftigung bei soliden Firmen in Branchen mit einem hohen Anteil an Zombie-Firmen geführt.

Jedoch müssen die Nebenwirkungen der unkonventionellen Geldpolitik nicht notwendigerweise problematisch sein, sondern können beispielsweise auch zu einer potenziell wünschenswerte Disintermediation von Unternehmenskrediten, d. h. Unternehmensfinanzierung durch Anleihenmärkte anstelle durch Banken, beitragen (Grosse-Rueschkamp et al. 2019).

Beim Unterschreiten der Nullzinsgrenze, also der Einführung von negativen Zentralbankzinsen und deren Transmission in den Bankensektor, besteht das Risiko, dass Einleger ihre Bankeinlagen abziehen, um durch Bargeldhaltung eine nominale Rendite von Null zu erreichen. Aus diesem Grund sind Banken tendenziell zögerlich, negative Zinsen, die Banken für ihre Zentralbankreserven zahlen müssen, an ihre Einleger weiterzureichen. Zwar haben Banken häufig die Möglichkeit, über Gebühren eine de-facto negative Verzinsung der Guthaben ihrer Einleger zu erreichen, aber die Überwälzung ist häufig imperfekt, da marginale Einlagen der Bank Kosten verursachen und Gebührenerhöhungen durch Wettbewerb begrenzt sind. Dies ist verstärkt im Wettbewerb mit Internetbanken, die vorteilhafte Kostenstrukturen aufweisen, der Fall. Insofern verringern Negativzinsen die Nettozinsmargen von Banken.

Dies gilt besonders für deutsche Banken, deren Geschäftsmodell im internationalen Vergleich durch besonders geringe Gebühreneinnahmen auffällt und daher vom Rückgang der Margen auf das Kreditgeschäft besonders betroffen sind (Dombret et al. 2019). Im OECD-Vergleich haben deutsche Banken in dem von den Autoren betrachteten Zeitraum nicht nur den niedrigsten Anteil von Gebühreneinkommen am Gesamteinkommen (25 % gegenüber einem Durchschnitt von 42 %), sondern darüber hinaus auch die höchsten Kosten am Gesamteinkommen (70 % gegenüber dem Durchschnitt von 60 %). Aus diesem Grund trifft deutsche Banken der Rückgang der Nettozinsmarge besonders hart. Die Problematik besteht darin, dass der Rückgang der Nettozinsmarge das Eigenkapital der Banken weiter reduziert, was deren Kreditangebot verringert. Beim Versuch, ihre Marge aufrecht zu erhalten um weiter ihre Kosten decken zu können, kann dies außerdem zu einer riskanteren Kreditvergabe führen (Heider et al. 2019).

Weiterhin besteht das Risiko, dass expansive Geldpolitik ab einer bestimmten Schwelle kontraktive Wirkungen entfaltet. Dies wird als Umkehrzinssatz (Reversal Interest Rate) bezeichnet. Der Umkehrzinssatz entsteht an der Schwelle, an der die indirekte Re-Kapitalisierung von Banken durch die Kursgewinne der festverzinslichen Wertpapiere in ihren Büchern die Verluste der durch die reduzierte Nettozinsmarge entgangenen Gewinne kompensiert. Da mit der Zeit die noch zum höheren alten Zinssatz festverzinslichen Wertpapiere fällig werden, steigt diese Schwelle im Zeitverlauf (Brunnermeier und Koby, 2018). Dieses Ergebnis unterstreicht, dass viele der Konsequenzen von Niedrigzinsen im Zeitverlauf möglicherweise an ökonomischer Signifikanz gewinnen.

Schließlich können Finanzstabilitätsrisiken auch außerhalb des Bankensektors auftauchen. Im vorherigen Abschnitt wurde diskutiert, inwieweit Renditen auf sicherere Anlageformen besonders stark gefallen sind, was impliziert, dass Risikoprämien auf riskantere Anlagen, wie beispielsweise Eigenkapital von Unternehmen, gestiegen sind. Unter der Voraussetzung einer minimalen Marktsegmentierung zwischen den Märkten für Eigen- und Fremdkapital kann es für Unternehmen profitabel sein, sich als marktübergreifender Arbitrageur bei den selber ausgegebenen Anleihen und Aktien zu engagieren. Ma (2019) zeigt Evidenz, dass Unternehmen bei einem nur geringen gesamtwirtschaftlichen Angebot an Staatsanleihen, was mit einer steigenden Risikoprämie einhergeht, verstärkt Kredite aufnehmen und die Krediterlöse in Form von Aktienrückkäufen ausschütten. Bei sinkenden risikofreien Zinssätzen besteht demnach die Gefahr, dass sich die Verschuldung und damit die Fragilität im Unternehmenssektor erhöht. Ebenso können durch potenzielles „Reach for Yield“-Verhalten von Seiten der Investoren (Becker und Ivashina 2015) besonders riskante Unternehmungen leichter an Kapital gelangen. Um potenziell negative Konsequenzen für die Finanzstabilität aufzufangen, wäre also eine verbesserte Finanzaufsicht wünschenswert^7^, beispielsweise indem Aufsichtskompetenzen auf die europäische Ebene gehoben und einheitlich hohe Standards etabliert werden, um den europäischen Binnenmarkt für Kapital zu vollenden (siehe auch Gnath et al. 2019). Ebenso ist es sinnvoll, Eigenkapitalfinanzierungen für Unternehmen attraktiv zu halten, um den Verschuldungsgrad und damit die Fragilität im Unternehmenssektor zu begrenzen. Da einige der problematischen Auswirkungen durch Niedrigzinsen auf Geldpolitik und Finanzstabilität durch einen unzureichend kapitalisierten Bankensektor verstärkt werden, wären vor allem bessere Eigenkapitalausstattungen^8^ von Banken ein wichtiger Fortschritt.

### Konsequenzen für die Realwirtschaft

Eine Sorge ist, dass Niedrigzinsen negative Konsequenzen für das bereits ohnehin geringe Produktivitätswachstum haben könnten. Durch eine weiter fallende Nachfrage nach Investitionskapital würde der Trend fallender Zinsen, in einer Art Teufelskreis, weiter bestärkt. Schwacher Wettbewerb trägt aber nicht nur zu niedrigen Zinsen bei, wie in einem obigen Abschnitt bereits diskutiert, sondern niedrige Zinsen können ebenso zu einer weiteren Schwächung des Wettbewerbs führen. So zeigen Liu et al. (2019), dass bei sinkenden Zinssätzen die Marktführer einer Branche besonders aggressiv konkurrieren. Aufgrund von Produktivitätsunterschieden zwischen dem Marktführer und den anderen Unternehmen einer Branche kann dies bei letzteren Anreize setzen, Investitionen zu reduzieren. Bei hinreichend niedrigen Zinssätzen dominiert dieser Wettbewerbseffekt den Investitionsanreiz der gefallenen Kapitalkosten und reduziert die Investitionen für die Gesamtbranche. Die Umverteilung von Marktanteilen zum Marktführer führt in diesem Szenario damit zu weniger Investitionen und Wachstum.

Den in den vorherigen Abschnitten betonten eher problematischen Auswirkungen von Niedrigzinsen zum Trotz senken fallende Zinsen die Kapitalkosten von Unternehmen und damit einen der wichtigsten Inputpreise. Dies erleichtert die Entschuldung von Unternehmen, was insbesondere im Nachgang der Finanzkrise von 2008 sowie der Euroschuldenkrise von 2010 für viele Unternehmen von hoher Wichtigkeit war. Zugleich verringern niedrige Zinsen die Investitionskosten. Dies ist für gesamtgesellschaftlich wichtige Zukunftsaufgaben von großem Vorteil, beispielsweise für die Transformation der Wirtschaft in Richtung Klimaneutralität. Auch in der jetzigen Coronavirus-Krise ist ein hohes relatives Kapitalangebot und damit einhergehende Niedrigzinssätze für Unternehmen von Vorteil, indem es fundamental solventen Unternehmen Möglichkeiten eröffnet, den durch die Pandemie ausgelösten Angebots- und Nachfrageschock durch relativ günstige Liquidität zu überstehen.

### Konsequenzen für Politik und Haushalte

**Figure 3:**
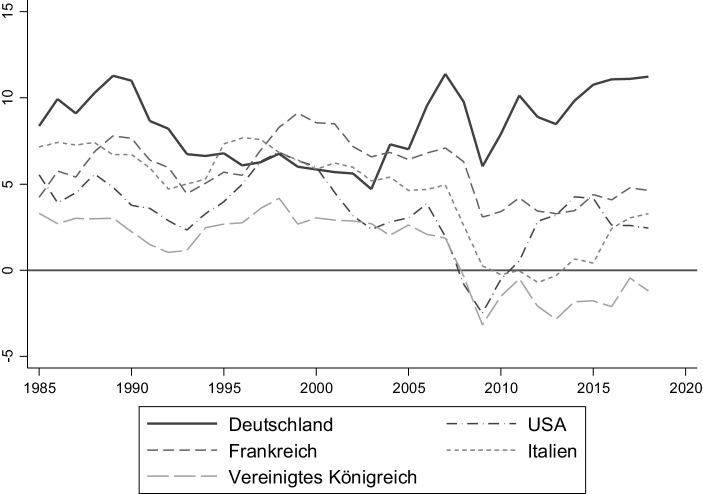

**Figure 4:**
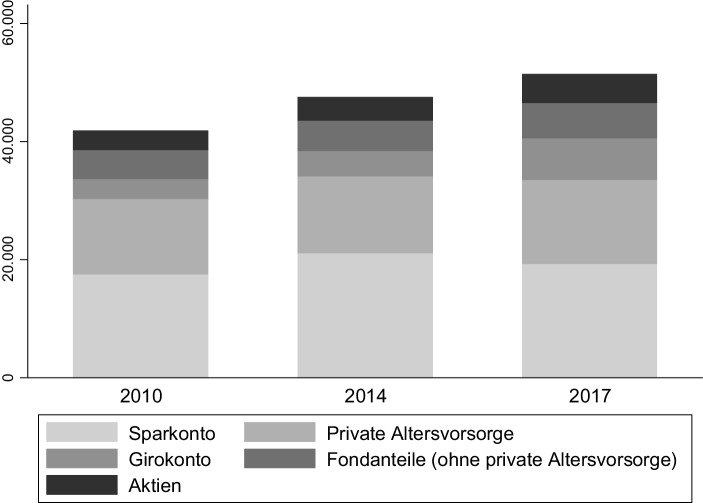

Deutsche Sparer sparen, anteilig am volkswirtschaftlichen Gesamteinkommen, seit vielen Jahren mehr als die meisten anderen entwickelten Ökonomien, siehe Abb. 3. Zugleich, wie in Abb. 4 ersichtlich, weisen deutsche Haushalte eine überdurchschnittlich hohe Präferenz für risikoarme Anlageklassen auf, wie beispielsweise Sparguthaben.

**Figure 5:**
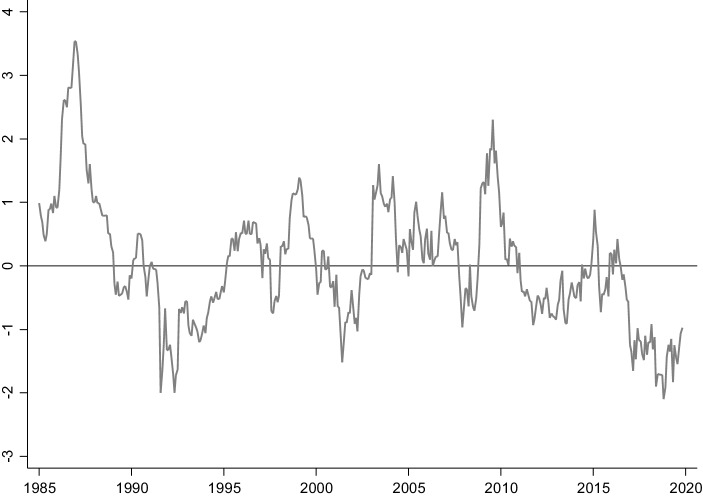

Aufgrund der starken Gewichtung dieser Anlageform in der deutschen Bevölkerung liegt das Hauptaugenmerk der deutschen Öffentlichkeit auf kurzfristigen nominalen Zinsen, insbesondere der Verzinsung von kurzfristigen Bankeinlagen. Diese liegen derzeit um Null. Aus Anlegersicht ist tatsächlich aber die Verzinsung nach Abzug der Inflation, d. h. die reale Verzinsung relevant. Wie in Abb. 5 erkenntlich, sind niedrige oder negativen reale Renditen auf sichere Anlagen mit kurzer Laufzeit (bspw. Bankeinlagen) keine neue Entwicklung, sondern historisch Normalität.

Der niedrige Ertrag auf diese Anlageformen ließe sich durch eine Umschichtung in riskantere und damit ertragreichere Anlageformen umgehen. Durch die fallenden Zinssätze sind die Preise vieler Vermögenswerte in den letzten Jahren stark gestiegen, was die sinkenden Renditen teilweise verschleiert hat. Zugleich haben deutsche Anleger aufgrund ihrer Untergewichtung von Immobilien und Aktien, siehe Abb. 4 und 6, von dieser Preisentwicklung nur wenig profitiert. Eine wichtige Konsequenz aus den niedrigen realen und nominalen Zinsen für den deutschen Sparer sollte also sein, seine Anlagepolitik anzupassen.

**Figure 6:**
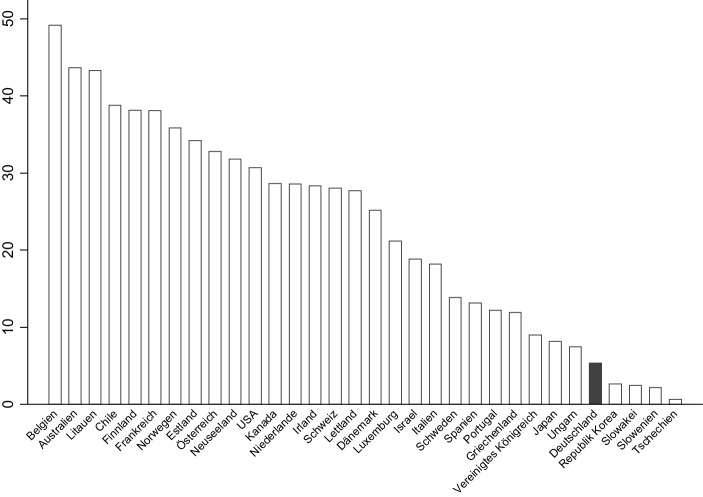

Eine der größten Herausforderungen durch dauerhaft niedrige reale Zinssätze ergeben sich bei der Altersvorsorge. Bei niedrigeren Zinssätzen wird es schwieriger, von bereits akkumuliertem Kapital zu leben. Dieser Effekt hat das Potenzial, den Lebensstandard von Pensionären, die ihre Einnahmen zumindest zum Teil aus einer kapitalgedeckten Altersvorsorge beziehen, zu reduzieren. Das kann zur Folge haben, dass sich die Akzeptanz der kapitalgedeckten Altersvorsorge in der Bevölkerung reduziert und politischer Druck entsteht, umlagefinanzierte Alterssicherungssystem auszubauen. Angesichts der durch eine demographisch bedingt wachsende Abhängigkeitsquote ebenfalls unter Druck geratenen umlagefinanzierten Sicherungssysteme wäre dies eine potenziell problematische Entwicklung.

Zugleich machen niedrige oder gar negative Zinssätze es ungleich schwieriger, ein bestimmtes Sparziel zu erreichen. Jüngere Beschäftigte, die für ihren Ruhestand sparen, müssen daher einen höheren Anteil ihres Einkommens zu sparen, um trotz des niedrigen Ertrags ihrer Anlagen einen angemessenen Lebensstandard im Ruhestand zu garantieren. Dabei sollten sie ihrem Anlagehorizont entsprechend vor allem in Aktien und Immobilien investieren.

Für ökonomische Akteure, sowohl Individuen, Unternehmen als auch den Staat, ergibt sich durch niedrige Zinssätze eine veränderte Entscheidungssituation bei Investitionsentscheidungen. Das trifft auf Staaten wie die Bundesrepublik Deutschland, die durch die Ausgabe von Schuldtiteln die hohe Nachfrage nach risikofreien Anlagen bedienen können, in ganz besonderer Weise zu. Investitionen, die unter anderen Finanzierungsbedingungen unprofitabel wären, können bei niedrigen Zinssätzen und damit niedrigen Diskontraten Sinn ergeben. Beispiele für solche Investitionsprojekte für Staaten sind Infrastrukturprojekte. Auch Unterstützungsprogramme für Unternehmen und Haushalte in der Coronavirus-Krise sind im Zeitalter sehr niedriger Renditen auf Staatsanleihen besonders lohnenswert.

Für Individuen können sich, auch vor dem Hintergrund der gestiegenen Nachfrage nach Humankapital, zusätzliche Studienjahre für eine bessere Qualifikation lohnen. Der Barwert höherer zukünftiger Einkommen ist größer als dies in einem Umfeld höherer Zinssätze der Fall wäre.

Bei sinkenden Finanzierungskosten sinken die relativen Preise von kapitalintensiven Gütern. Als kapitalintensivstes Konsumgut wird dies im besonderen Maße Wohnraum betreffen nach dem eine erhöhte Nachfrage zu erwarten ist. Zugleich steigen jedoch die Preise von Vermögenswerten, die nicht im beliebigen Ausmaß vermehrt werden können. Dies trifft auf Grund und Boden zu, besonders auf Grundstücke in beliebten Lagen. Um einen zusätzlichen Anstieg des Bodenwertes und den sich daraus ergebenden Bodenrenten zu verringern kommt der Stadtplanung daher eine besondere Bedeutung zu, die insbesondere in den Großstädten durch eine weitere Bereitstellung von Bauland für eine Nachverdichtung sorgen sollte.

## Schlussfolgerung

Abschließend lässt sich zusammenfassen, dass für die fallenden Zinssätze eine Reihe von Ursachen in Frage kommt. Wenngleich die wissenschaftliche Debatte um die Bestimmung der Ursachen noch nicht abgeschlossen ist, sehen die Autoren dieses Beitrags die in der deutschen Öffentlichkeit häufig verbreitete Auffassung, dass der Hauptverursacher der niedrigen Zinssätze die EZB sei, als nicht haltbar an. Dafür sprechen vor allem die globalen und langfristigen Eigenschaften der fallenden Zinssätze bei gleichzeitig niedriger Inflation. Die Evidenz deutet stattdessen auf Ursachen struktureller Natur hin, wie in den vorhergehenden Abschnitten dargelegt. Auch wenn das relative Gewicht der Argumente umstritten bleibt, gibt es nur wenige Gründe, davon auszugehen, dass die Niedrigzinsphase in naher Zukunft überwunden sein wird. Ebenso ist der Einfluss der durch das Coronavirus ausgelösten Krise zu diesem Zeitpunkt noch völlig unklar. Insofern ist es wichtig, für politischen und ökonomische Entscheidungen die richtigen Schlussfolgerungen zu ziehen und diese in den Entscheidungsprozess mit einzubeziehen. Aus den zuvor diskutieren Punkten ergeben sich aus Sicht der Autoren dieses Beitrags unter anderem die folgenden Konsequenzen:

1. Weitere Stärkung der Eigenkapitalbasis von Banken.
2. Ausbau der gebührenbasierten Geschäftsmodelle von Banken zulasten ihrer traditionellen Zinsmargengeschäfte bei gleichzeitiger Kostenreduktion.
3. Stärkung der Finanzaufsicht.
4. Erhöhung der Attraktivität von Eigenkapitalfinanzierung für Unternehmen, beispielsweise durch eine steuerliche Gleichbehandlung von Fremd- und Eigenkapital.
5. Stärkung des Wettbewerbsrechts und besserer Durchgriff der Wettbewerbsaufsicht, insbesondere mit Blick auf die Plattformökonomie.
6. Umschichtung der Portfolios von privaten Anlegern in Aktien.
7. Verstärktes Sparen der jüngeren Generationen, ihrem Zeithorizont entsprechend vor allem in renditeträchtigere Anlagen.
8. Bessere Bereitstellung von Bauland in Großstädten und deren Umgebungen.
9. Vorsichtige Ausweitung der Staatsverschuldung Deutschlands zur Finanzierung von langfristigen Investitionen; gerade vor dem Hintergrund der Coronavirus-Krise ist besonders bei niedrigen Renditen auf Staatsanleihen ein großzügiges staatliches Unterstützungsprogramm möglich und sinnvoll.

## Footnotes

1 Siehe beispielsweise Wiebe und Narat (2019).

2 Bei Carry-Trades (dt. Zinsdifferenzgeschäfte) zwischen zwei Währungspaaren wird ein Kredit in der Währung mit dem niedrigen Zinssatz aufgenommen und in der Währung mit dem höheren Zinssatz angelegt. Durch das Schaffen von Nachfrage nach Krediten in der Niedrigzinswährung und Angebot von Krediten in der Hochzinswährung übt dieses Geschäft zugleich Aufwärtsdruck auf den niedrigeren und Abwärtsdruck auf den höheren Zinssatz aus.

3 Dies gilt für am Markt gehandelte Risiken. Nicht arbitragefähig sind u. a. durch Informationsasymmetrien getriebene Aufpreise, z. B. Kompensation für das „Monitoring“ von Bankkrediten oder die Kosten externer Finanzierungsquellen gemäß der Hackordnungstheorie.

4 Der gewichtete Durchschnitt der Eigen- und Fremdkapitalkosten ist unter der Abkürzung WACC bekannt.

5 Beispielsweise durch Verwendung der Marktrisikoprämie bei der Anwendung des CAPM.

6 Tatsächlich relevant ist die sog. „effektive Nullzinsgrenze“, die mit negativen Nominalzinsen einhergehen kann. Sie ist durch die Lagerkosten von Bargeld nach unten begrenzt.

7 Der Betrugsfall der Wirecard AG zeigt deutlich den Reformbedarf.

8 Der Ausstattung der Banken durch Bail-in-fähiges Fremdkapital zur Vermeidung von Bankenrettungen durch Steuermittel ist ebenfalls von hoher Bedeutung. Allerdings spielen besseren Eigenkapitalpuffern aufgrund positiver Effekte auf das Kreditangebot, z. B. bei der wirtschaftlichen Erholung nach einer Krise, eine besondere Rolle (Jorda et al. 2020).
